# Passive Acoustic Tracking of Singing Humpback Whales (*Megaptera novaeangliae*) on a Northwest Atlantic Feeding Ground

**DOI:** 10.1371/journal.pone.0061263

**Published:** 2013-04-10

**Authors:** Joy E. Stanistreet, Denise Risch, Sofie M. Van Parijs

**Affiliations:** 1 Northeast Fisheries Science Center, Woods Hole, Massachusetts, United States of America; 2 Duke University Marine Laboratory, Beaufort, North Carolina, United States of America; 3 Integrated Statistics, Inc., Woods Hole, Massachusetts, United States of America; University of Manitoba, Canada

## Abstract

Passive acoustic tracking provides an unobtrusive method of studying the movement of sound-producing animals in the marine environment where traditional tracking methods may be costly or infeasible. We used passive acoustic tracking to characterize the fine-scale movements of singing humpback whales (*Megaptera novaeangliae*) on a northwest Atlantic feeding ground. Male humpback whales produce complex songs, a phenomenon that is well documented in tropical regions during the winter breeding season, but also occurs at higher latitudes during other times of year. Acoustic recordings were made throughout 2009 using an array of autonomous recording units deployed in the Stellwagen Bank National Marine Sanctuary. Song was recorded during spring and fall, and individual singing whales were localized and tracked throughout the array using a correlation sum estimation method on the time-synchronized recordings. Tracks were constructed for forty-three song sessions, revealing a high level of variation in movement patterns in both the spring and fall seasons, ranging from slow meandering to faster directional movement. Tracks were 30 min to 8 h in duration, and singers traveled distances ranging from 0.9 to 20.1 km. Mean swimming speed was 2.06 km/h (SD 0.95). Patterns and rates of movement indicated that most singers were actively swimming. In one case, two singers were tracked simultaneously, revealing a potential acoustic interaction. Our results provide a first description of the movements of singers on a northwest Atlantic feeding ground, and demonstrate the utility of passive acoustic tracking for studying the fine-scale movements of cetaceans within the behavioral context of their calls. These methods have further applications for conservation and management purposes, particularly by enhancing our ability to estimate cetacean densities using passive acoustic monitoring.

## Introduction

Studies of animal movement require a means of reliably locating an animal as it moves through the surrounding environment. For many species, direct observation of movement in the field is impractical or impossible, especially in marine environments. For several decades, very-high frequency (VHF) radio telemetry systems were the primary method of tracking individual animal movement, and were used in both marine (e.g. [1]–[3]) and terrestrial (e.g. [4]–[6]) habitats. The development of modern satellite telemetry and GPS technologies has stimulated major advances in animal tracking [7], and these methods are currently widely applied across taxa, including birds (e.g. [8]), mammals (e.g. [9]), and fishes (e.g. [10]). Nonetheless, limitations remain, including the need to attach transmitters directly to animals, which may affect behavior [11], and the high cost per unit, which frequently limits the number of individuals studied [12]. Passive acoustic localization offers an alternative method of tracking fine-scale animal movements, where an array of time-synchronized acoustic sensors is used to estimate the position of a sound source within the array. Although this method is restricted to vocally active animals that pass through or near an array, it is unobtrusive and can potentially be used to track a large number of individuals without the increased cost and effort of attaching individual transmitters.

Passive acoustic localization has been of primary interest in the study of cetaceans [13], [14] and other marine mammals (e.g. [15], [16]) due to their reliance on sound as a primary modality for communicating underwater, as well as the challenges of observation in the marine environment. Passive acoustic methods have been used to address a wide variety of ecological and behavioral questions, such as identifying the calling individual (e.g. [17], [18]), attributing recorded sounds to a particular species (e.g. [19], [20]), estimating density and distribution of animals (e.g. [21], [22]), understanding reproductive strategies (e.g. [15], [23]), and studying the impacts of anthropogenic ocean noise on animal behavior (e.g. [24], [25]).

Localization of cetacean sounds can be accomplished using either mobile or fixed hydrophone array configurations. Hydrophone arrays towed behind ships are increasingly used in conjunction with visual cetacean surveys to improve estimates of animal abundance (e.g. [21], [26]). Acoustic localization of vocal individuals and groups from the survey vessel allows the matching of recorded sounds to visual sightings [19] and the estimation of an animal's trajectory relative to the vessel [27]. Mobile acoustic systems such as towed arrays provide flexibility in spatial coverage, but are limited in duration by cost and the considerable effort in operating survey vessels. Fixed arrays, which typically consist of autonomous or cabled hydrophones moored to the seafloor, are constrained to a specific geographic area, but allow the collection of longer-term (seasonal, annual, and multi-annual) datasets [13]. For consistently vocalizing animals, detailed tracks of movement can be constructed from acoustic position estimates as frequently as every few minutes. Fine-scale movement information from passive acoustic tracking has been used to study behavioral responses of cetaceans to anthropogenic noise disturbance from ships [28] and seismic airguns [25]. Similarly, passive acoustic tracking provides opportunities to study the ecology of cetaceans in the context of their vocal behavior. For example, Hastie et al. [29] used a vertical array to track the diving behavior of bottlenose dolphins, and determined foraging depths by localizing the call types associated with prey capture events. Calls thought to be associated with reproductive behavior, such as the songs produced by males of some baleen whale species (e.g. [30], [31]), may also provide behavioral context for an animal's observed movements.

In our study, we used passive acoustic tracking with a fixed hydrophone array to characterize the fine-scale movement patterns of singing humpback whales (*Megaptera novaeangliae*). Humpback whales are highly vocal, and males produce long, repetitive song sequences [30]. Most humpback whales perform annual migrations between lower-latitude winter breeding grounds and higher-latitude summer feeding grounds [32]. Song is produced by adult males, and regularly occurs in the tropics during the winter breeding season [33], [34]. Humpback whale song has also been reported along migration routes and within higher-latitude regions (e.g. [35]–[37]). Previous studies have incorporated acoustic tracking with visual survey methods to document the movements of singing males and their interactions with females and non-singers. Most of this research has been conducted on tropical breeding grounds [38]–[40], and to a lesser extent along migration routes [41], [42]. A recent study by Stimpert et al. [43] documented the underwater dive behavior and concurrent song production by two tagged humpback whales in Antarctic waters. To our knowledge, no other data exist describing the movements of singers on feeding grounds. Year-round passive acoustic monitoring in the Stellwagen Bank National Marine Sanctuary (SBNMS), a northwest Atlantic feeding ground for humpback whales, has allowed song occurrence in this region to be quantified across seasons, and facilitated the tracking of singers using passive acoustic localization.

The objectives of our study were twofold: (1) to demonstrate the use of passive acoustic tracking for characterizing fine-scale movements of cetaceans; and (2) to provide an initial description of the movements of singing humpback whales in the northwest Atlantic outside their traditional breeding season.

## Methods

**Figure 1:**
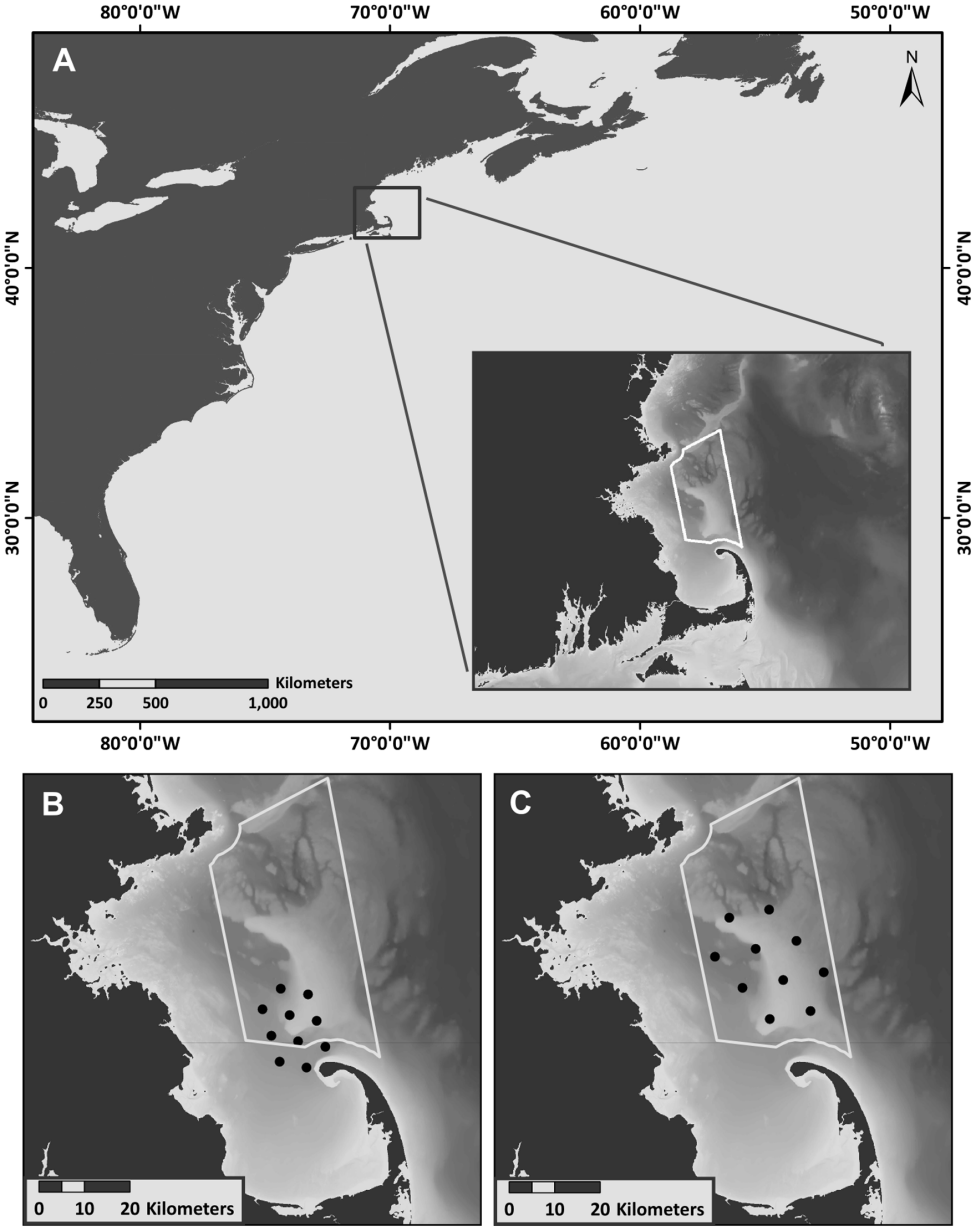
Map of the study location in the northwest Atlantic ocean. The inset shows Stellwagen Bank National Marine Sanctuary outlined in white (A). Black dots indicate the locations of bottom-mounted Marine Autonomous Recording Units deployed from (B) 28 March to 28 May 2009, and (C) 2 October to 15 December 2009. Bathymetry map provided by M. Thompson, Stellwagen Bank National Marine Sanctuary.

Our study was conducted within the Stellwagen Bank National Marine Sanctuary (SBNMS), located in the southern Gulf of Maine in the northwest Atlantic Ocean (42° 33′, N 70° 25′ W; Fig.1a). The sanctuary encompasses an area of 2,180 km^2^ and features a shallow, sandy bank and rich biological productivity, making it an important feeding habitat for several cetacean species. Humpback whales typically frequent SBNMS between April and December to feed on sand lance (*Ammodytes* spp.) and other small schooling fishes [44]. Long-term passive acoustic monitoring over multiple years has shown that humpback whale song regularly occurs on the SBNMS feeding ground from April through May, following the spring migration, and from August through December, preceding the fall migration [45].

### Data Collection

Continuous acoustic recordings were made using arrays of Marine Autonomous Recording Units (MARUs), as part of a 3-year research project focused on large-scale monitoring and mapping of underwater noise within SBNMS [46]. MARUs are bottom-mounted archival recording units, developed by Cornell University's Bioacoustics Research Program (www.birds.cornell.edu/brp/hardware/pop-ups; [47]). Each unit consists of an HTI-94-SSQ hydrophone attached to a pressurized glass sphere containing computer electronics, batteries, and a hard drive. Each hydrophone had a sensitivity of −168 dB *re* 1 V/*μ*Pa and was connected to a 23.5 dB preamplifier. The frequency response was flat (±1 dB) over the 10–585 Hz frequency range. MARUs were programmed to record continuously at a sampling rate of 2000 Hz and a 12-bit resolution.

Our study used data from arrays of ten MARUs deployed for consecutive three-month periods throughout 2009 (Fig. 1b & 1c). Individual units in each array were placed 3 to 6 nautical miles apart, and the arrays were shifted seasonally within SBNMS to target areas with high cetacean concentrations at different times of the year. The depth of units varied from 24 m to 130 m, dependent on bathymetry. In each array, all MARUs were time-synchronized immediately before deployment and upon retrieval to allow correction for any slight clock drift occurring in individual units. Recordings were time-aligned and compiled into 10-channel data files. Spectrographic analysis was conducted using the eXtensible BioAcoustic Tool (XBAT; [48]) run in MATLAB 7.4.

### Seasonal Song Occurrence

Initially, the yearlong data set was examined for the presence of humpback whale song. Continuous 24-hour recordings were made on 361 days in 2009; the remaining 4 days (13 March, 28 May, 2 October, 15 November) lack complete recordings due to the time needed to retrieve and deploy MARUs; data from these dates were not included. Source levels for humpback whale song have previously been measured at 151 to 173 dB *re* 1 *µ*Pa [49], and song has been recorded over ranges as far as 29 km from the source within our study region, using similar equipment [47]. When present in 2009, humpback whale song was generally heard across multiple MARUs, and consequently SBNMS was considered to be a single acoustic area. To quantify seasonal song occurrence, a single MARU was chosen from each array, from a location to the north or east of Stellwagen Bank in order to facilitate comparison to datasets from previous years (see [45] for details). Hourly song presence was assessed using an automated template detector based on spectrogram cross-correlation. Detector performance was evaluated using identical methods to those described by Vu et al. [45], and a false negative detection error rate of 10% was estimated for the 2009 dataset. All hours with detections were viewed by a human analyst to confirm song presence. False positive detections were removed, and hours with no detections were not reviewed, since the 10% rate of missed hours with song was considered acceptable for this analysis.

### Movement Analysis

**Figure 2:**
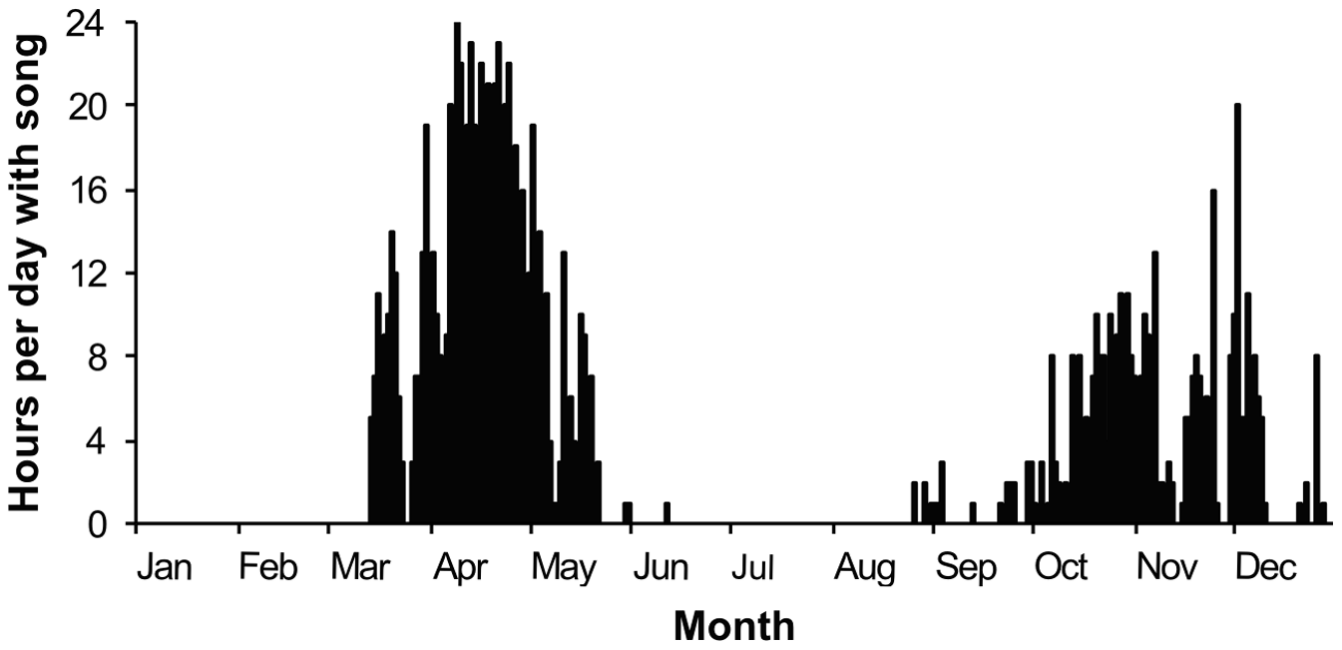
Occurrence of humpback whale song within Stellwagen Bank National Marine Sanctuary throughout 2009. Recordings were made across 12 months, and song occurrence is represented as the number of hours per day during which song was detected.

Individual singing humpback whales were acoustically located and their movements tracked during the spring and fall peaks in song occurrence identified in Fig. 2. Song sessions were chosen for localization based on song session duration, clarity of sound recorded across multiple channels, and position of the singer within the array. Only periods of song lasting 30 minutes or longer, clearly visible on spectrograms of three or more channels, and produced by a singer within the acoustic array were used.

Within each song session, individual song units were selected for localization and a two-dimensional (x, y) position was computed for each selection using the correlation sum estimation (CSE) tool [50] developed for XBAT. The CSE location algorithm uses an iterative process to determine the potential source location of a selected sound within a horizontal plane. The sum of waveform cross-correlation values across all channel pairs are calculated for a set of points in space, and the point that maximizes this sum is selected as the most likely location. Unlike traditional localization algorithms in which the source location is calculated using cross-correlation peaks to generate geometric hyperbolae and solve for the intersection point, this method requires no *a priori* decisions to be made regarding location and time delays, and is considered robust to background noise ([50], see also [51]). Each resulting location was reviewed to ensure that the correct song unit was chosen on all channels during the cross-correlation process, and that the estimated location agreed with the observed time of arrival differences across channels. Song units for which reasonable locations could not be obtained were removed from the dataset, ensuring that tracks did not contain any major outliers.

Locations were computed every 60–120 seconds for the duration of each song session, as long as the singer remained within the acoustic array. Gaps of 2 to 5 minutes between consecutive locations occasionally occurred when song became faint or was briefly inaudible in the recordings (e.g., when the singer surfaced to breathe). A few longer gaps occurred when the signal-to-noise ratio on one or more channels was too low to compute locations, usually due to increased background noise from passing ships. Acoustic localization produced a set of positions with xy coordinates and associated times for each analyzed song session.

The CSE algorithm did not generate estimates of localization uncertainty; therefore a calibration experiment was conducted to determine location error. A series of frequency-modulated “sweep” tones were played on 27 March 2009 and 28 May 2009 at known locations and depths within the array. After the acoustic data were retrieved, these sweeps were identified in the sound files and locations were estimated using the CSE algorithm. Error was calculated by measuring the difference in meters between the acoustically estimated location and the known source location. Mean localization error was 53.2 m (SD 30.76), calculated from 47 sweep tones played at five different locations within the array.

**Figure 3:**
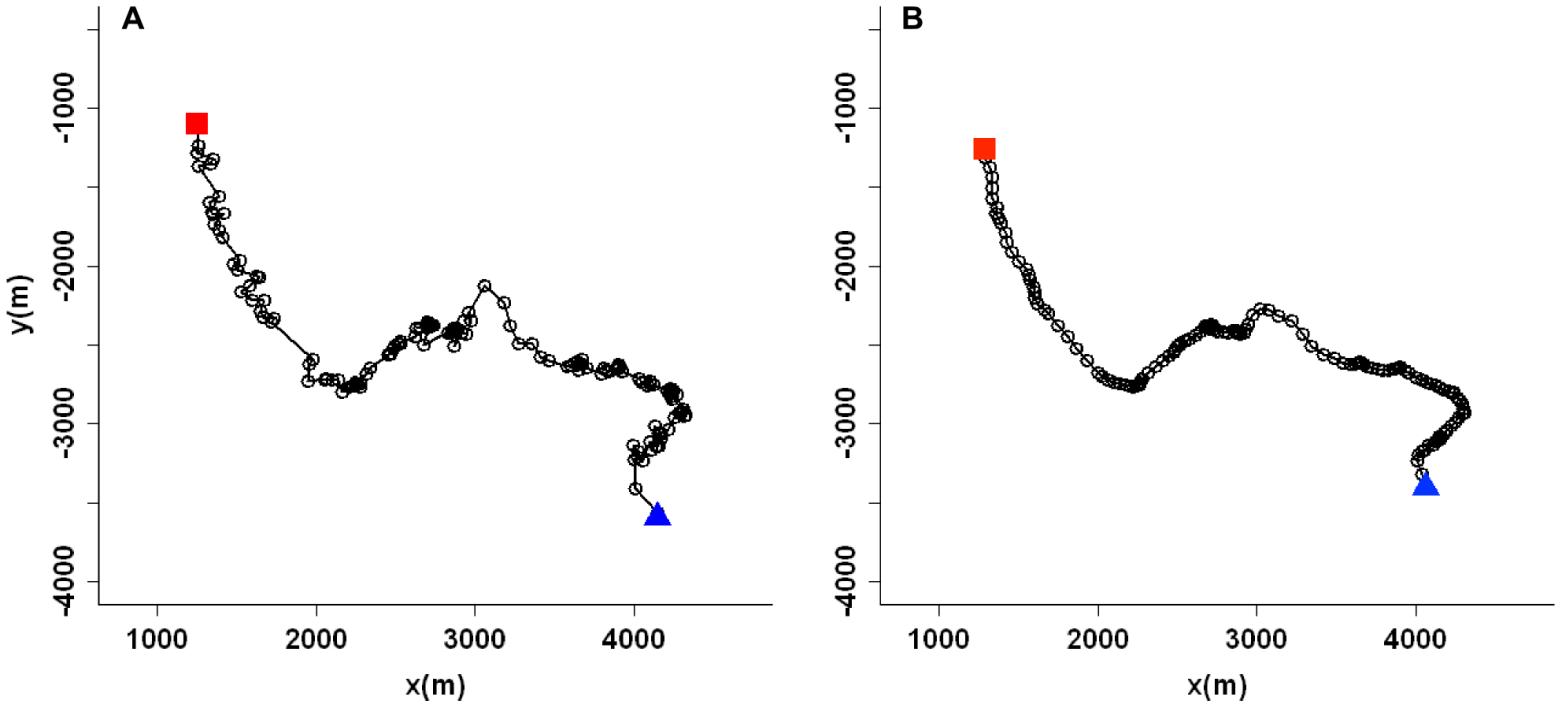
Effect of the smoothing function on the acoustic track of a singing humpback whale. Track is shown before (A) and after (B) applying a moving average smoothing function to the estimated locations (5-point window, centered at median time).

For each localized singer, a “track” was defined as the complete, time-ordered collection of locations from a single song session, with moves between locations represented as straight lines [52]. Prior to analyzing track characteristics, raw tracks were smoothed using a moving average (MA) technique. To calculate the smoothed location at time *t*, the average of the surrounding five locations centered at *t* was computed [53]. At the cost of a slight reduction in time resolution for each track, smoothing reduced the “wiggle” due to location imprecision (Fig. 3), and allowed a more realistic estimation of average swimming speed and distance traveled over the length of the track. The MA smoothing technique was chosen to reduce the influence of localization error on movement parameters measured at the whole-track scale, and was considered appropriate for this analysis since manual review of the localization process prevented large localization errors within tracks.

Track characteristics were analyzed using the *adehabitat* package [54] within the R software version 2.12.1 [55]. This package provides various functions for the analysis of animal trajectories, including the calculation of basic movement descriptors. Each track was analyzed as an *ltraj* class object [56], which calculates the distance, time interval, net displacement, and turning angle for each step of the trajectory, where a step represents the straight-line move between two sequential locations. Stepwise measurements were summed to examine four parameters at the whole-track level: duration (h), net displacement (km), total distance traveled (km), and average speed (km/h). Directedness of travel of each singer's path was quantified using a straightness index (SI), defined as the net displacement from start to end divided by the total distance traveled [57]. An individual traveling in a straight line will have a SI value of 1, while an increasingly meandering path will result in a SI value closer to 0. While the smoothing function increased SI values by reducing total track length, the smoothed tracks likely provided a more realistic representation of each whale's movement. The SI was used only to quantify the major differences in directedness of travel at the whole-track scale, not at the finer scales on which the smoothing function was implemented. In addition to descriptive statistics for each track parameter, non-parametric Spearman's rank correlation coefficients were computed for pairwise comparisons of all track parameters.

### Multiple Singer Example

In one instance it was possible to track two singers simultaneously within 5 km of each other. Movement patterns were examined in detail for both singers to reveal changes in movement behavior potentially resulting from an acoustic interaction. Distance between the two singers was measured when the period of overlapping song began and ended. Each singer's track was separated into two segments: an “alone” period when the whale sang individually, and a “both” period when the two whales sang simultaneously. Duration, net displacement, distance traveled, speed, and straightness index were calculated for each track segment.

### Ethics Statement

This research was conducted with permission from the Stellwagen Bank National Marine Sanctuary. No animals were approached during this study, and no permits were required for passive acoustic data collection or instrument calibrations, which complied with all relevant regulations.

## Results

### Seasonal Song Occurrence

Humpback whale song was detected during 149 of 361 recording days in 2009. All detected song occurred within two distinct periods (Fig. 2). During spring, song occurred from 14 March through 11 June, with a marked peak in singing activity in the middle of April, and an overall mean of 8.7 (SD 7.55) hours per day with song. During the fall, song was detected from 26 August through 28 December, with a less pronounced peak in late November, and an overall mean of 3.3 (SD 3.96) hours per day with song. No song was recorded during either the summer months (mid-June to mid-August) or winter months (January to mid-March). Song occurrence was significantly higher during the spring than fall based on mean number of hours per day with song (Welch's t-test, t = 6.16, *df* = 123.3, *p*<0.001).

### Movement Analysis

**Figure 4:**
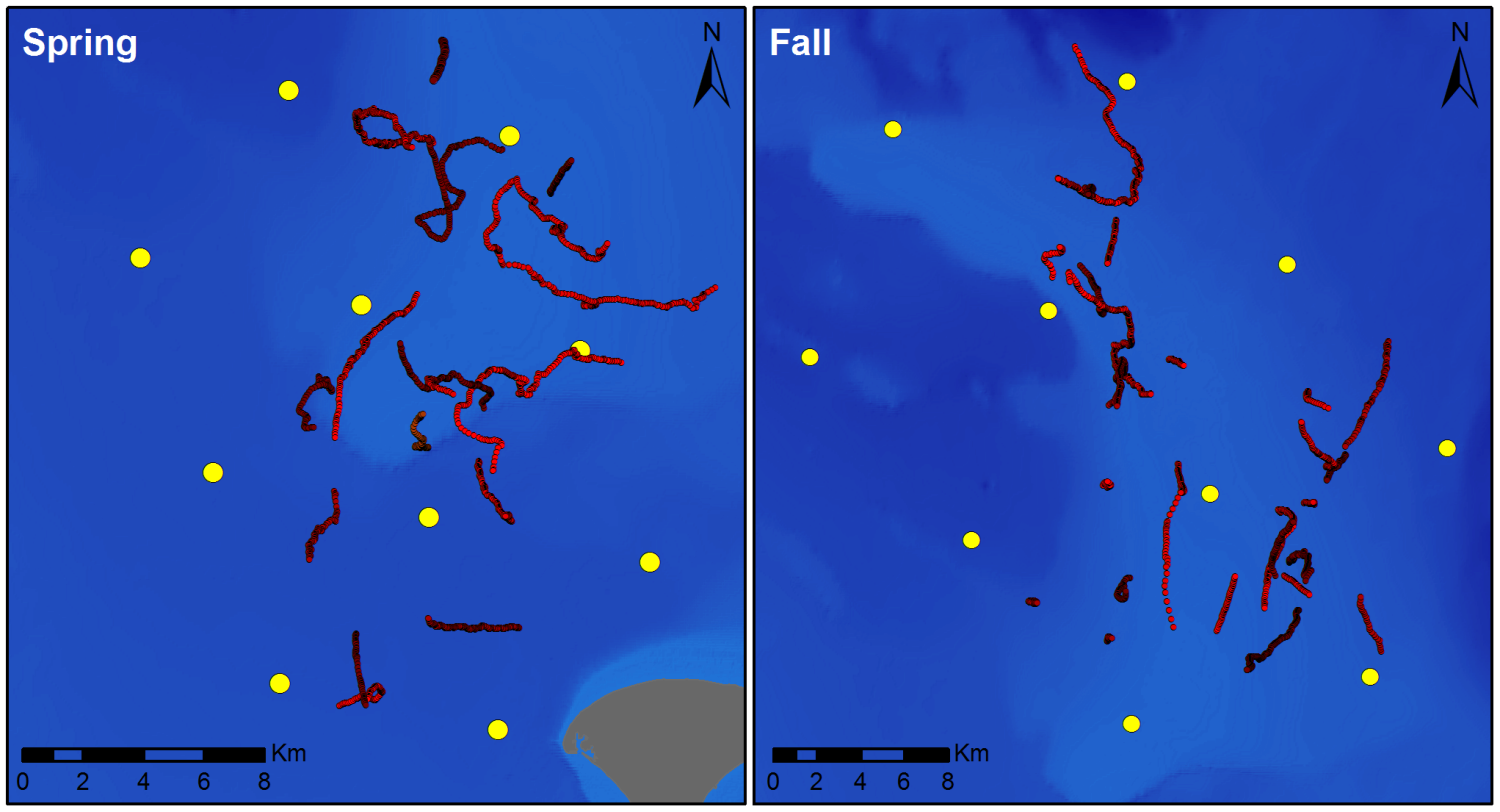
Maps of the acoustic tracks of singing humpback whales recorded during spring and fall 2009. Yellow dots indicate the arrays of bottom-mounted Marine Autonomous Recording Units used for acoustic recording, localization, and tracking. Whale tracks are shown in red. Maps include all singing whales tracked during the spring and fall study periods; tracks did not occur simultaneously. Bathymetry map provided by M. Thompson, Stellwagen Bank National Marine Sanctuary.

Forty-three song sessions were tracked during the study: 17 (total of 47.7 h) between 1 April and 3 May 2009, and 26 (total of 49.6 h) between 25 October and 26 November 2009 (Fig. 4). To improve the likelihood of including different individuals in the analysis, we selected song sessions spaced throughout the approximately 30-day study period surrounding the peak in song occurrence during each season. Multiple singers were sometimes heard simultaneously during the study period, indicating that more than one male was present within detection range of the acoustic array. However, not all singing whales were tracked, since many did not meet the criteria previously outlined in our methods.

**Figure 5:**
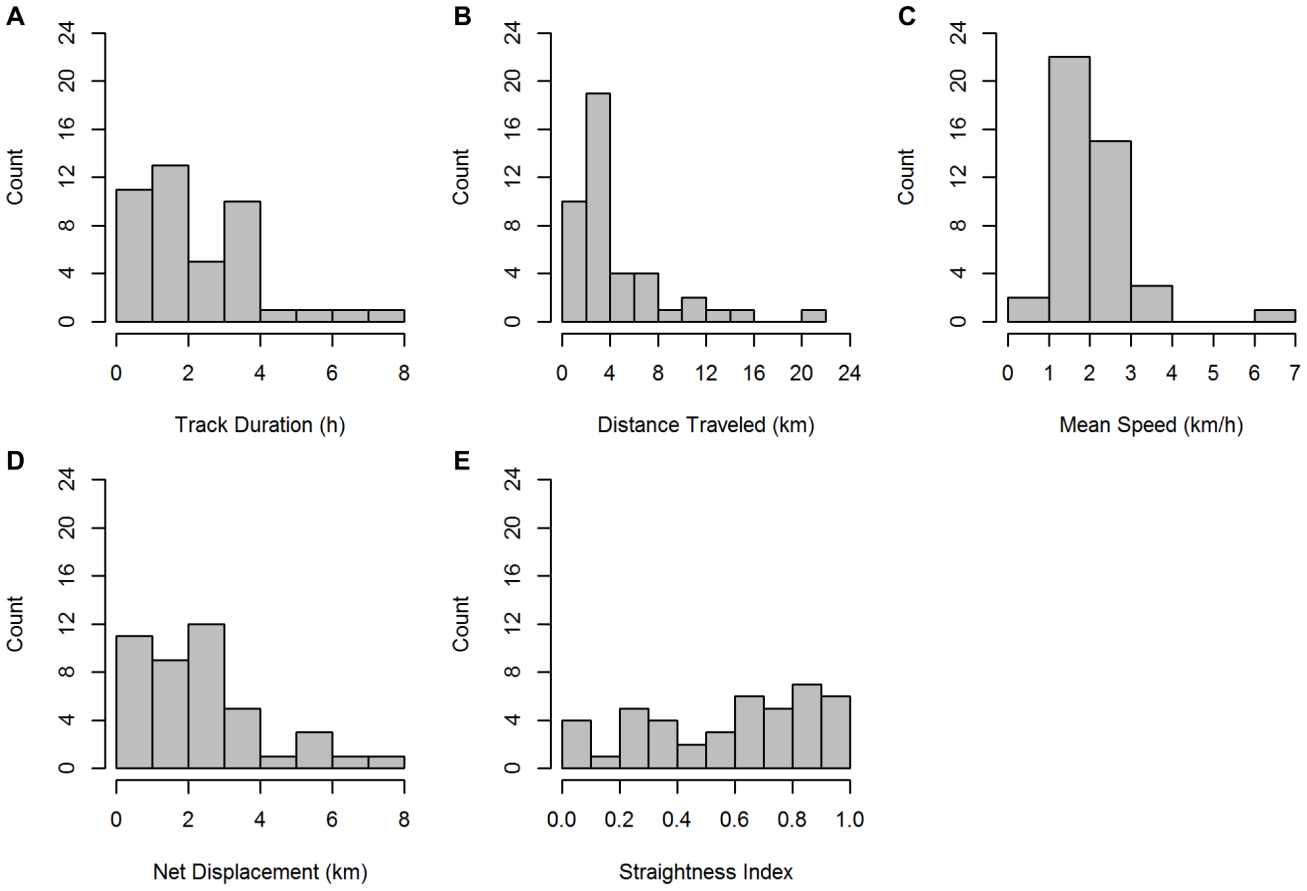
Frequency histograms calculated from the acoustic tracks of *n* = 43 singing humpback whales. Histograms show the distributions of each measured track parameter: track duration (A), total distance traveled (B), average swimming speed, calculated as the total distance traveled divided by the track duration (C), net displacement (D), and straightness index (E).

A high level of variation was observed among individual tracks (Fig. 4 & Fig. 5). Track duration ranged from 0.46 to 7.69 h (mean 2.26 h; SD 1.67) (Fig. 5a). All tracked singers exhibited some degree of movement while singing, traveling distances between 0.69 km and 20.13 km (mean 4.46 km; SD 3.98) (Fig. 5b). Average speed, calculated from track duration and distance, ranged from 0.54 to 6.05 km/h, with a mean of 2.06 km/h (SD 0.95) across all tracks (Fig. 5c). The smoothing function should minimize the effect of location imprecision, which could falsely inflate calculated speeds, and provide more conservative estimates of swimming speeds. The directionality of movement varied considerably between tracks, ranging from highly meandering, with a small net displacement and a straightness index close to 0, to highly directional, with a net displacement similar to the total distance traveled, and a straightness index close to 1 (Fig. 5d & 5e). Smoothing likely increased straightness index values for all tracks, but allowed this index to quantify major differences in directedness of travel without being strongly influenced by finer-scale location imprecision.

**Figure 6:**
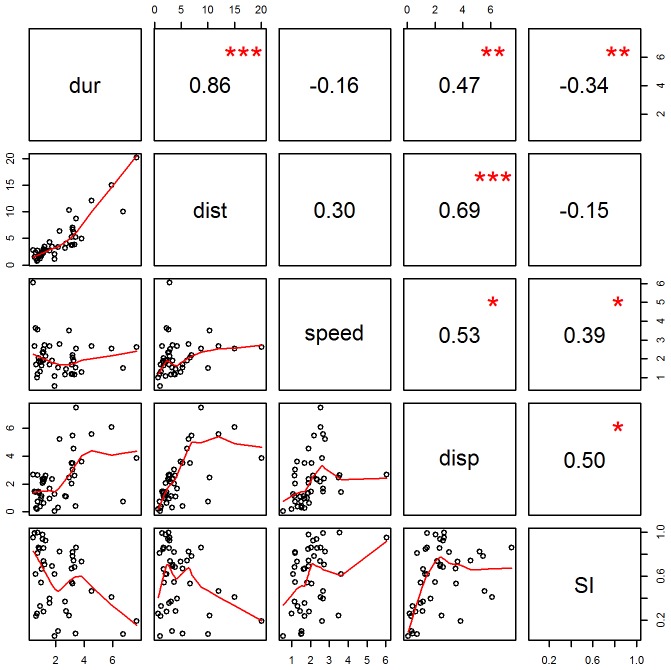
Correlation matrix with pairwise comparisons of track parameters. Parameters are labeled along the diagonal: dist  =  distance traveled (km), dur  =  duration (h), speed  =  average speed (km/h), disp  =  net displacement (km), SI  =  straightness index (unitless). Scatterplots are shown in the lower left triangle below the diagonal; Spearman's rank correlation coefficients are shown in the upper right triangle above the diagonal. Red asterisks indicate significance level: ***<0.001, **<0.01, *<0.05.

Average speed was positively correlated with both straightness index and net displacement (Spearman's rank correlation, p<0.05; Fig. 6), indicating that singers traveling in a straight path tended to swim faster than those traveling along a more meandering path. In addition, straightness index was negatively correlated with track duration (Spearman's rank correlation, p<0.01; Fig. 6), indicating that movement paths were straighter during short song sessions. Strong positive correlations between track duration, distance traveled, and net displacement were also found; these results are not surprising given that whales are likely to exhibit a greater degree of movement over increasing periods of time.

A summary of mean track parameters by season is provided in Table 1. Similar seasonal means for spring and fall were calculated for average speed and straightness index. Although track duration, distance traveled, and net displacement were slightly higher during the spring than fall, no significant differences were found between the spring and fall seasons in any of the parameters measured (t-tests, *df* = 41, p>0.05 for each test), due to the high variation among tracks within seasons.

**Table 1:** Comparison of summary statistics for the acoustic tracks of singing humpback whales recorded during the spring and fall seasons.

	Duration (h)	Total distance (km)	Average speed (km/h)	Net displacement (km)	Straightness index
**Spring (n = 17)**					
Mean±SD	2.81±1.96	5.68±4.91	2.01±0.70	2.42±1.47	0.55±0.26
Minimum	0.85	1.59	1.07	0.73	0.07
Maximum	7.69	20.13	3.49	5.58	0.93
**Fall (n = 26)**					
Mean±SD	1.91±1.38	3.66±3.09	2.09±1.09	2.19±1.93	0.60±0.31
Minimum	0.46	0.69	0.54	0.06	0.05
Maximum	5.92	14.99	6.05	7.50	1.00

Spring tracks were recorded between 01 April 2009 and 03 May 2009; fall tracks were recorded between 25 October 2009 and 26 November 2009. Mean, standard deviation (SD), and range (minimum & maximum) are reported for measured track parameters within each season.

### Multiple Singer Example

**Figure 7:**
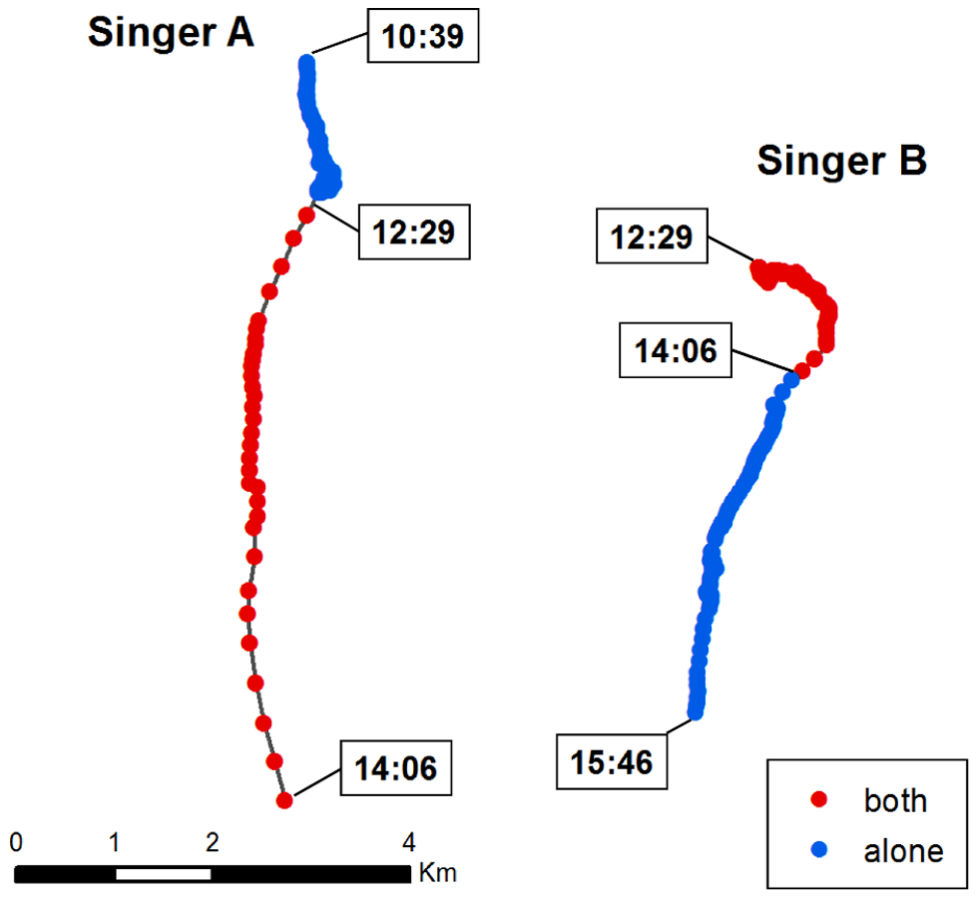
The acoustic tracks of two singing humpback whales over a 5 h time period. Song occurred simultaneously for 1.5 h. Blue points indicate the locations of each individual when singing alone; red points indicate the locations of singers while both songs were recorded simultaneously. Times (in EST) when each song session began and ended are indicated on both tracks.

On 17 November 2009, a singing whale was tracked in the southern region of the MARU array (Fig. 7, Singer A). Song began at 10:39 EST, and the singer moved in a generally southward direction at an average speed of 1.4 km/h. At 12:29, song from a second individual (Fig. 7, Singer B) was recorded, located 4.6 km to the east. At this time, Singer A began to move southward on a more directional path, with average speed increasing to 3.8 km/h during the period of overlap when both songs were recorded. A straightness index of 0.52 before and 0.96 during the period of song overlap was calculated for Singer A. The movement of Singer B was slower (average speed 1.3 km/h) and less directional during the period of overlap, and generally oriented in a south/east direction. After 1.5 hours, song from Singer A became more intermittent, and locations were obtained less frequently, although the singer remained within range of the acoustic array. A final position was estimated at 14:06 before this song ceased. The two singers were 6.7 km apart at this time. After Singer A ceased singing, the average speed of Singer B increased to 2.5 km/h and movement became more directional, oriented southward and slightly to the west until song ceased at 15:46. The straightness index of Singer B increased from 0.50 during the period of song overlap to 0.90 while Singer B sang alone.

## Discussion

Research on humpback whale song and associated male behavior has largely been focused on the tropical breeding areas where the highest proportion of singing occurs (e.g. [38], [40], [58]). Our study extends this work to higher latitudes in the northwest Atlantic, providing a description of the movement patterns exhibited by singing whales on a feeding ground.

In 2009, humpback whale song was prevalent in Stellwagen Bank National Marine Sanctuary (SBNMS) during the spring and fall, with the highest occurrence of song recorded from mid-March to mid-May. Overall this pattern is similar to the annual song occurrence described by Vu et al. [45] for the same study area in two prior years, 2006 and 2008. While there is some year-to-year variation in off-peak song occurrence during the summer and winter months, the spring and fall peaks in singing occur in the same months and to a similar extent each year. Our results from 2009 provide additional evidence that the occurrence of humpback whale song in this region follows a consistent annual pattern, with a sharp increase in song during the pre- and post-migration periods. Humpback whales do not leave SBNMS during the summer period of low song occurrence from June to September, but cease singing while feeding [45]. In contrast, few individuals remain in the area during the winter period of low song occurrence between January and March [59].

Forty-three song sessions were acoustically tracked within SBNMS during the spring and fall of 2009, demonstrating highly variable movement patterns across both seasons. While some of the observed movement may be due to passive drift with currents, the rates of travel and frequent changes in direction occurring within tracks generally indicated active swimming. Earlier work conducted on lower-latitude breeding grounds described singing whales as lone and relatively stationary or slow-moving [33], [60]. Frankel et al. [38] provided evidence for active swimming by singers in Hawaii, using acoustic localization to estimate a mean swimming speed of 1.6 km/h (*n* = 31). Along Pacific migration routes off the east coast of Australia, Noad & Cato [41] calculated a mean swimming speed of 2.45 km/h for *n* = 57 singing whales, and reported a few instances of much higher sustained swimming speeds, up to 7.0 km/h maintained for more than an hour. Mean swimming speed measured in our study (2.06±0.95 km/h) was slightly higher than the breeding ground estimate by Frankel et al. [38], and comparable to the mean speed observed during migration by Noad & Cato [41]. Our speed estimates were averaged over each whole track after smoothing, and changes in speed during a song session were not accounted for. Partial-track speeds likely exceed the minimum and maximum whole-track speeds reported here. Most previous studies describing the fine-scale movements of singing humpback whales have been conducted in the Pacific, and the breeding behavior of the western North Atlantic population is less well described in the literature. Swimming speeds have not been reported on Atlantic breeding grounds using passive acoustic localization of singers. However, Whitehead & Moore [61] examined movement patterns by visually following whales at the surface, and described the movements of singers on Silver Bank as “slowly meandering” with mean speeds between 0.5 and 1.5 knots (0.9 and 2.8 km/h).

Clark and Clapham [47] suggested that song production occurring outside the typical breeding season may be hormonally-driven, and recent studies have further discussed this hypothesis [45], [62]. It has been suggested that elevated testosterone levels in advance of the fall migration and residual levels remaining after the spring migration may contribute to the production of song at higher latitudes. However, it remains unknown whether singing males are engaging in intra-sexual displays and/or courtship of females in this region. The high rates of movement and variable movement patterns observed in SBNMS may be related to the low density of humpback whales in this area during the spring and fall. Although we lack density estimates for SBNMS during the study period, broad-scale aerial surveys throughout the Gulf of Maine region have demonstrated low sighting rates during April and November when song occurrence peaks (<0.02 whales/n mile surveyed; [59]). Singers may therefore be moving about in search of conspecifics, whether females and/or other males. By contrast, peak densities of up to 1.25 whales/km^2^ (4.3 whales/n mile^2^) have been reported on Silver Bank and Navidad Bank during the height of the winter breeding season [61]. Whitehead & Moore [61] additionally reported that singers on Silver Bank preferentially display in smooth-bottom areas. Depth and bottom type may be factors contributing to the movement of singers within SBNMS, which contains heterogeneous bottom features (Fig. 4). Since the distribution of tracks in our study was determined largely by the acoustic localization range of a spatially-restricted hydrophone array, we were not able to relate singer locations to different bathymetric features. A broader survey of singers throughout a larger region may reveal bottom-type preferences.

While breeding and feeding behaviors in humpback whales were traditionally thought to be spatially and temporally separated, several studies have documented song in feeding areas at various times of year [35]–[37], [45]. Stimpert et al. [43] recently documented foraging by singing humpback whales near the Western Antarctic Peninsula in the austral fall season. The use of multi-sensor tags allowed the dive profiles and feeding lunges of tagged whales to be analyzed during periods when song was recorded. Results suggested that humpback whales on this Antarctic feeding ground may switch back and forth between foraging and breeding/display behavior when food is present. While we cannot discern feeding behavior from our two-dimensional track data, we did not find evidence of singers remaining in a single area for a prolonged time. The observed movements of singing males may indicate that singers are actively searching for food and/or feeding sporadically during song sessions. Further studies using behavioral tags, combined with other methodologies such as passive acoustic tracking and visual observation, could provide more insight into this behavioral flexibility and the tradeoff between feeding and song production.

Acoustic tracking methods are constrained by their ability to provide information only on vocally active individuals, and interpretation of the observed movement patterns in the context of social behavior requires information on other individuals present, including non-singing males and females. However, when multiple singers are recorded simultaneously, acoustic tracking can provide a means of examining singer interactions. Previous studies have used acoustic localization to measure separation distances between two or more singing males [38], [40], [63]. Frankel et al. [38] hypothesized that song may function to maintain spacing between males, and provided evidence of avoidance behavior among singers, which maintained greater separation distances than non-singing whales. In contrast, Cholewiak [63] tracked multiple singers and found that singing males approached each other more often than expected by chance, sometimes performing “close approaches” of less than 1 km, which often led to the cessation of song by one of the singers. Extensive observations by Darling et al. [40] of singer interactions with other singing and non-singing adults further suggested that song may function to mediate a variety of interactions between males. The multiple singer example described here illustrates a potential acoustic interaction between two singing males: Singer A demonstrated faster, more directional movement during the period of song overlap with Singer B, as compared to the preceding period when singing alone. In addition, Singer B's movement pattern underwent a similar change after song from Singer A ceased, suggesting that the movements of Singer B may also have been modified by the presence (or absence) of Singer A. The two singers in this example did not approach each other while both were singing, and were separated by a distance of several kilometers, which increased during the period of song overlap. While male-male interactions are highly variable and likely depend on the context and identities of the individuals involved [40], further examples could help elucidate the types of singer interactions occurring in this region.

Humpback whales are ideal for study using passive acoustic tracking, as they produce loud, patterned song sessions that allow individuals to be consistently tracked. However, these methods are applicable to other cetacean species as well (e.g. [25], [64]), and provide opportunities to collect information on movement patterns that may be difficult or costly to obtain using individual transmitter tags. Statistical methods and tools for analyzing animal movement data are undergoing rapid development as GPS and satellite tracking technologies improve [7]. Although passive acoustic tracking has a different set of limitations and considerations than movement data collected from individual satellite or GPS transmitters, similar analytical methods can be applied once a time-ordered set of geographic positions has been obtained. Software packages such as *adehabitat* provide a suite of functions for movement analysis (see [54] for an overview), which have been used to examine habitat selection (e.g. [65]), home ranges (e.g. [66]), migratory patterns (e.g. [67]), and other behavioral and ecological processes related to the movements of individuals.

Passive acoustic localization and tracking methods have further applications for conservation and management efforts. Understanding the geographic distributions and population sizes of cetacean species are critical goals, particularly since many species are threatened by human activities. Mobile passive acoustic surveys have been combined with traditional visual line-transect surveys to generate improved abundance estimates [21], and methods for estimating density of cetaceans using fixed passive acoustic sensors are rapidly being developed [22], [68], [69]. Various approaches have been described using fixed arrays (e.g. [22]) and single moored hydrophones (e.g. [68]), but a remaining source of variance in density estimation models is introduced by the necessity to link the rate of detected calls to the number of calling animals present. For many species, information on calling rates of individuals and groups is largely unknown, and may depend on group size, behavioral context, time of day, or other variables [22]. As demonstrated here, passive acoustic tracking can be an effective means of studying the behavior of calling individuals, and may provide further species-specific and context-dependent information for estimating the population densities of cetaceans.
